# Book Reviews

**Published:** 1898-02

**Authors:** 


### Book Reviews.

THE DISEASES AND INJURIES OF THE CONJUNCTIVA, ESPECIALLY THE SO-CALLED GRANULATED LIDS. By Dr. Jno. H. Thomson, Professor of Ophthalmology and Otology, Kansas City Medical College, Kansas City, Mo.

In this era of rush and hurry, of prolonged seasons of physical and mental activity and severe nervous strain, every professional man looks about him for the easiest and simplest methods of providing himself with the best "tools of his trade."

In the field of medicine this is particularly true. Every physician can not be a specialist, yet each must have a general knowledge of every branch of his profession. A recent publication from the Hudson Kimberly press, of Kansas City, Mo., will meet a long-felt want in the way of a treatise on diseases of the eye.

The little volume, one of whose chief claims to favor is its convenient size, makes no extravagant claims for itself.

In the preface, the author, Dr. Jno. H. Thomson, of the Kansas City Medical College, states it is a series of essays that have proven to be of value to the general practitioner, in aiding him to recognize and treat diseases of the conjunctiva, granulated lids, etc. The little volume is one that will be of practical service to its readers.

---

OUTLINES OF RURAL HYGIENE—FOR PHYSICIANS, STUDENTS AND SANITARIANS. By Harvey B. Bashore, M. D., Inspector for the State Board of Health of Pennsylvania. With an Appendix on the Normal Distribution of Chlorine by Prof. Herbert E. Smith, of Yale University. Illustrated with twenty (20) engravings. 5½x8 inches. Pages vi–84. Extra cloth, 75 cents net. The F. A. Davis Co., Publishers, 1914-16 Cherry St., Philadelphia; 117 W. Forty-Second St., New York City; 9 Lakeside building, 218-220 S. Clark St. Chicago, Ill.

In these days of preventive medicine, the study of hygiene and sanitary science is of paramount importance. The growth and prosperity of a city is largely dependent upon the care taken of the health of its inhabitants, but neglect of prophylaxis in the country is notorious. If those whose practice is confined to the rural districts would see fit to inform themselves on matters of practical sanitation and domestic hygiene they would become powers in the land whose influence would be seen in the suppression of infectious disease.

The little book before us contains just such information as is needed on the subject of water supply, waste disposal, habitations, disposal of the dead, and is to be heartily recommended to country practitioners and their patrons as well.

LECTURES ON THE MALARIAL FEVERS. By William Sidney Thayer, M. D., Associate Professor of Medicine in the Johns Hopkins University. New York. D. Appleton & Co. 1897.

A glance at the medical literature of the past few years, at the many and conflicting opinions as given in the medical journals, and at the mortuary statistics, is sufficient to impress one with the necessity of a thorough and comprehensive work on the subject of the malarial fevers.

This we are fortunate in now having, from the pen of Dr. Thayer, in a book of 315 pages.

The treatment is most thorough and includes descriptions of methods of examination of the blood, of the various forms of the malarial parasite, of the clinical forms of the disease, complications, morbid anatomy, diagnosis and treatment.

The differential diagnosis between the malarial fevers and some of the diseases with which it is most commonly confused is also taken up and discussed.

The work is abreast with the latest scientific discoveries, but at the same time, coming from the pen of one of large experience with the disease, is entirely practical.

The work will be of the greatest value to all practitioners in regions where malaria is met with, and should be in the library of every physician, wherever he may be living.

---

MANUAL OF PATHOLOGY. By W. M. Late Coplin, M. D., Philadelphia. P. Blakiston, Son & Co. 1897.

In the preface the author states that he "wishes to submit the volume, not as a treatise or book of reference, but, as its title indicates, as a manual which he hopes may be useful in the laboratory, post-mortem room, and in clinical diagnosis by the aid of the microscope."

In this difficult task of condensing in a single volume of medium size the principles of pathology and the technique of laboratory methods, the author has been remarkably successful. Part I. is devoted to technique and treats of post-mortem examinations, histological methods and the examination of urine, sputum and blood. Part II. is on general pathology, and includes chapters on bacteriology and animal parasites. Part III. contains eleven chapters on special pathology and covers the important structure and organs. The treatment of the various subjects is clear, concise and abreast with the latest scientific investigations.

The illustrations are numerous, and in execution much superior to those usually seen.

We predict for this work an early recognition of its excellence and its adoption in many schools as a text-book.

A MANUAL OF GYNECOLOGY. By Henry T. Byford, M. D., Chicago. Second edition, containing three hundred and forty-one illustrations, many of which are original. Philadelphia. P. Blakiston, Son & Co., 1012 Walnut street. 1897.

The second edition of this well-known work contains much new material. Separate parts have been added on the anatomy of the pelvic organs, venereal diseases, angioma, hypertrophy and subinvolution of the uterus; also chapters on urinary and fecal fistulæ, and ectopic gestation besides the use of the cystoscope, urethral instruments and matters of minor detail. A feature of value is the system of marginal notes, given as pointers to the important things in each paragraph. The work is profusely illustrated and the text is unusually clear. The whole book is characterized by extreme clearness and conciseness, few words being wasted on what is not strictly germain to the subject, so that as a guide for instruction in this important branch of medicine and surgery it is sure to prove of the greatest value.

---

ELEMENTS OF LATIN. For Students of Medicine and Pharmacy. By George D. Crothers A. M., M. D. Teacher of Latin and Greek St. Joseph (Mo.) High School. Formerly Professor of Latin and Greek, University of Omaha; and Hiram H. Bice, A. M., Instructor in Latin and Greek Boys High School, New York. Philadelphia, New York, Chicago. The F. A. Davis Company, 1898.

Some acquaintance with Latin and Greek, while not essential, is of great assistance to the student of medicine and pharmacy. It greatly facilitates the proper understanding of a large number of the terms used in medicine and allied sciences and removes one and a not inconsiderable obstacle in the way of the student. This book constitutes an easy course, but full enough to give a student a very fair working knowledge. The words selected for conjugation and declension and in the exercises are those which occur in the province of medicine and botany. To these is added a list of anatomical proper names and their origin, and also a general vocabulary. To all who desire to become well-rounded in the profession of medicine, and who have lacked the opportunity of thorough preliminary education, we would suggest that they apply themselves to the study of Latin, which they will find an agreeable and profitable pastime. To such, this volume will be admirably suited.

---

ABOUT CHILDREN. Six Lectures given to the Nurses in the Training School of the Cleveland General Hospital in February 1896. By Samuel W. Kelley, M. D., Prof. Diseases of Children in the Cleveland College of Physicians and Surgeons. The Medical Gazette Publishing Co., Cleveland, Ohio. 1897.

This little book consists of a collection of a series of lectures delivered by the author and is especially designed for the use of nurses and all those who have the care of children. There is much in it that addresses itself to a more critical audience—the medical profession, and the practical treatment of the subjects considered can not fail to appeal commendably to it.

The book is written and published by a doctor and for that reason, if for no other, deserves the support of the profession. That it is entitled to such on its own merits, a perusal of it will quickly demonstrate. There will be found a great many little practical points that are apt to be overlooked in the text-books, but which are so important in the proper conduct of a case and which must be learned by experience and observation unless recourse be had to a work of this character. Dr. Kelley appreciates this need and has given most acceptably his counsel and advise.

